# Effectiveness of the tailored Evidence Based Practice training program for Filipino physical therapists: a randomized controlled trial

**DOI:** 10.1186/1472-6920-14-147

**Published:** 2014-07-17

**Authors:** Janine Margarita R Dizon, Karen Grimmer-Somers, Saravana Kumar

**Affiliations:** 1 International Centre for Allied Health Evidence, University of South Australia, City East Campus, North Terrace, Adelaide 5000, Australia; 2 University of Santo Tomas, Manila 1015, Philippines

**Keywords:** EBP, Training, Physical therapists, Developing country, Knowledge, Skills, Attitudes, Behavior

## Abstract

**Background:**

This study evaluated the effectiveness of the contextualized EBP training program for Filipino physical therapists in terms of knowledge, skills, attitudes and behavior.

**Methods:**

A randomized controlled trial was designed to assess the effectiveness of the EBP training program. Fifty four physical therapists were randomly allocated to the EBP group (intervention) and waitlist (control) group. The EBP group had a one day face-to-face training with an online support, whilst the control was waitlisted. There were three measurement points which were pre, post, and three months post intervention for knowledge, skills and attitudes. Activity diaries were used to measure behavior. The diaries were collected after three months. Data analysis was by intention to treat in EBP domains of knowledge, skills and attitudes.

**Results:**

Fifty-four physical therapists were included in the study. Fifty two (52) completed the post training assessment and 26 completed the 3 months post training assessment for EBP knowledge, skills and attitudes. There were significant improvements in these domains in the EBP group from pre to post training and over a period of three months (p < 0.05) compared with the waitlist control group. Thirty seven (37) physical therapists completed their activity diaries over three months. Behavior significantly improved in the EBP group in terms of EBP behaviors (formulating PICO, searching, appraising and applying the evidence) when faced with both new/unique and usual case scenarios (p < 0.05). More physical therapists in the waitlist control group significantly performed non-EBP behaviors (asking doctors and reading textbooks) when faced with new/unique cases compared with the EBP group (p < 0.05). No differences were noted between groups regarding non-EBP behaviors (asking colleagues and doctors and reading textbooks) particularly when faced with usual cases.

**Conclusion:**

The contextually designed EBP training program for Filipino physical therapists was effective in improving knowledge, skills and attitudes to EBP from pre to post training. Improvements were also observed at three months post training in knowledge, skills, attitudes and behavior to EBP. This model of training can be modified as needed based on the needs of the local context. Findings need to be interpreted with caution due to study limitations.

**Current controlled trials:**

ISRCTN74485061 (Registration date: February 9, 2011).

## Background

Achieving best patient outcomes and optimising use of health resources should be the goal of every health system, whether in developed or developing countries. The importance of using evidence to guide practice in health care has been on the international agenda for some 20 years [1]. Evidence-based practice (EBP) has become an integral component of healthcare and strategies to ensure that health care is underpinned by the best evidence are well established in most developed countries [2]. Moreover, in the developed world, training for health professionals to put evidence into practice (Evidence-based practice (EBP) programs) is widely available and regularly evaluated at undergraduate and post graduate levels, and for continuing professional development [3-5]. The evidence for many health interventions for the management of a range of conditions have been summarized, and made accessible for the use of health professionals, policy makers and patients [6-9]. Increasingly common are the development, implementation and evaluation of clinical guidelines based from the evidence and present recommendations for practice [10,11].

In developing countries, evidence-based practice is in its infancy. Whilst the principles of EBP are particularly relevant in developing countries to underpin health policy and health delivery, the growth of EBP has been slower in developing countries. Some reasons for the slow growth are lack of knowledge of, and skills in, EBP, limited to lack of evidence-base information on developing country conditions, lack of access to electronic databases, limited research capacity, and lack of time to do EBP related activities [12-14]. Developing countries are also faced with more challenging concerns than developed countries regarding evidence-informed practice. These concerns are largely based on queries regarding the usefulness and relevance of EBP in local contexts [12,15-20]. Differences in practice, the nature of health systems, scarce health care and research resources and other local and context specific conditions, further constrain the relevance and applicability of EBP in developing countries. For instance, in the Philippines, the nature of physical therapy practice is different compared to other countries. Physical therapists in the Philippines are not first contact practitioners and are given a prescribed treatment for the patients by referring doctors who have specialized in rehabilitation medicine. This influences and at the same time challenges the way they think and treat patients [21].

Whilst there may have been efforts to introduce EBP into developing countries, there is no clear or practical approach to building capacity and promoting the use of EBP in local settings that results to improved behaviors [16-18]. With limited resources available (financial, facilities, manpower) and the cultural and contextual issues confronting individual developing countries, it is unlikely that health professionals would invest their money, time and efforts in learning and adapting the principles of EBP unless they are perceived to be relevant and useful to their practice or their patients, in their context.

This paper presents the findings of research into a model of an EBP training program designed to build capacity and promote EBP among physical therapists in a developing country, the Philippines. The EBP training program in this study has been considered as a ‘complex intervention’ based on the Medical Research Council of the United Kingdom (MRC) definition of complex interventions and informed by the stages in MRCs framework [22]. Complex interventions consist of multiple phases and components and each phase informs the next phase of the intervention [22]. To address the ‘complexity’ of complex interventions, methods to standardize these types of interventions were recommended by identifying the ‘fixed/constant’ and ‘variable’ components [23]. ‘Fixed’ components are regarded as the *essential functions,* while the ‘variable’ components are those that fit the needs and the context of the population and the local setting where they operate. The concept of standardizing complex interventions into fixed and variable components, particularly in conducting EBP training, has been proposed in the literature [24] and applied to this model of EBP training. The ‘fixed’ components of the EBP training were composed of the concepts and steps to EBP whilst the ‘variable’ components were composed of the EBP Checklist (to make recommendations to referring doctors to support the use of evidence-based management), the online support, use of printed materials and more time for the practical sessions as the physical therapists need to be guided appropriately [21].

The objectives of this study were:

1. To assess the effectiveness of the contextually-based EBP training program in improving knowledge, skills, attitudes and behavior of Filipino physical therapists; and

2. To provide a tested model of continuing education and uptake of evidence relevant to Filipino physical therapists.

## Methods

The detailed methods and study procedures underpinning this randomized controlled trial were published as a full study protocol [21]. A summary of the study methods is provided in this paper, for completeness.

### Ethics

This study had ethics approval by the University of South Australia’s Human Research Ethics Committee (protocol no. 0000021872). Written informed consents were formally obtained from the participants who took part in this study.

### Design

A randomized controlled trial was conducted, in which physical therapists from the Philippines were randomly allocated to an EBP group or a waitlist control group using computer generated random numbers. Assessments of EBP outcomes occurred at three time periods: pre-training, immediately post-training and three months after the training. Activity diaries were completed by both groups during the three month follow up period, during which time the EBP group also received support in terms of the EBP online support and an EBP checklist to recommend treatment interventions.

### Sample

The participants were physical therapists in the Philippines recruited from a range of sources: a database of physical therapists obtained from a preliminary descriptive survey study [25,26], the network of the Philippine Physical Therapy Association and a list of hospitals in the yellow pages (telephone). Potential participants were screened for the following criteria:

1. Licensed physical therapists and

2. No previous formal EBP training.

#### Sample size

The sample size for this study was calculated from the findings of the pilot RCT [27] using Medcalc software. The primary outcome measure used as basis for sample size calculation was knowledge and skills using the Adapted Fresno Test. The pilot RCT resulted to a large effect size (0.80) which required only a sample of 10 participants. As we are uncertain what inflated the effect size, we decided to be conservative and used a moderate effect size (0.4-0.7) to compute for the sample size at 0.05 level of significance and with an 80% statistical power. Fifty four (54) physical therapists were then required for this study.

#### Randomization and blinding

Once participants were screened for eligibility, an independent person randomly allocated participants into the EBP intervention group and the waitlist control group, using computer generated random numbers applied to the list of eligible participants. Allocation was concealed from the researchers of this study and to the assessors. The schedule of training was dependent on the group allocation such that the EBP intervention group was scheduled ahead and those in the control group were waitlisted, and provided with the training after the three month period of data collection has been completed. Those assessing the outcomes of the study were blinded at all times.

#### Development of the intervention

Preliminary studies were undertaken prior to this RCT to inform the development of the program and to identify specific strategies to enhance the delivery of the program to the participants. The EBP training program was entitled the ‘EBP for FilPTs’. The training program was modelled as a complex intervention with “fixed/constant’ and ‘variable’ components. It was also underpinned by theories of adult learning [28], educational strategies [29-32] and the evidence regarding the effectiveness of EBP training programs [33]. It was then designed in the context of the needs of the physical therapists and the nature of the local practice [25,26] considering that they are not first contact practitioners.

### ‘Fixed’ components

The ‘fixed’ component of the EBP for FilPTs is the one day face-to-face training, in the form of lectures and practical sessions, consisting of the following lectures with supporting practical sessions (to influence skills in EBP):

1. Introduction to EBP

2. Hierarchy of evidence and study designs

3. Drafting the clinical question using the PICO format

4. Designing the search

5. Critical appraisal of the evidence and

6. Answering the clinical question based from the evidence found.

#### ‘Variable’ components

The ‘variable components of the program are the EBP Checklist, the online EBP support, use of printed materials and more time for practical sessions. The EBP Checklist (Additional file 1) is to make evidence-based recommendations to referring doctors. It contains items related to validity and applicability of the evidence, magnitude of effects and capacity of the physical therapist to deliver the evidence-based patient management. The online support can be accessed through http://unisa.edu.au/Research/Sansom-Institute-for-Health-Research/Research-at-the-Sansom/Research-Concentrations/Allied-Health-Evidence/Quality-Care/EBPPhil/The-EBP-Training-Package and is for the use of physical therapists who lack time to search, and lack access to evidence based information. Physical therapist would also have access to the training materials (lectures and references) in case they need to refresh their knowledge regarding the EBP [21,25-27].

### Outcome measures

#### EBP knowledge and skills

The Adapted Fresno Test (AFT) was used to measure changes in EBP knowledge and skills. It is reported to be a psychometrically-sound test especially among novice allied health professionals [34]. The AFT is composed of clinical scenarios relevant to physical therapy and occupational therapy and items related to formulating a question, identifying and searching for sources of evidence, identifying the best study type to answer the question and relevance, validity and significance of the evidence found. The AFT is scored using a scoring rubric and the total possible score is 156. The use of the AFT for this RCT was tested in the pilot RCT conducted prior to this study, where it was assessed for reliability (ICC = 0.99) between two assessors [27].

#### EBP attitudes

The questions regarding EBP attitudes developed and used by Stevenson, Lewis and Hay (2004) [35] were applied to measure changes in attitudes among the physical therapists in this study. These were questions related to changing practice if good quality evidence exists, support to undertake EBP activities, and confidence in searching the literature and undertaking critical appraisal. The questions are to be answered by choosing whether the physical therapists agree, disagree or neither. This has been validated for content among physical therapists in the United Kingdom, and was found to be a valid measure of EBP attitudes [35].

#### EBP behavior

Behavior regarding EBP was measured by activity diaries. The physical therapists in both groups were instructed to log their activities by placing a tick (√) in the options related to activities used to find the answer to a case they were faced with. The case could relate to a unique situation or to usual care. A unique case has been defined as a new one for the physical therapist, or a difficult or complex case such as a patient with Parkinson’s disease who also had co-morbidities, making the condition complex. A usual case has been defined as a common case such as non-specific low back pain where there are multiple possible treatment interventions, and where the best intervention needs to be identified. The physical therapists were also instructed to write a short description of the case in the diaries.

### Data analysis

Data were analysed using SAS version 9.2. For equal interval data, the findings were reported as medians, interquartile range and confidence interval for the median. For categorical data, findings were reported as percentages. Differences between groups were calculated using the Mann Whitney U test for interval data and Chi square test of proportion for categorical data. For all testing, p values were deemed to be significant if p < 0.05. Data analysis was by intention to treat for EBP knowledge, skills and attitudes but not possible for behavior.

## Results

### Study sample

**Figure 1:**
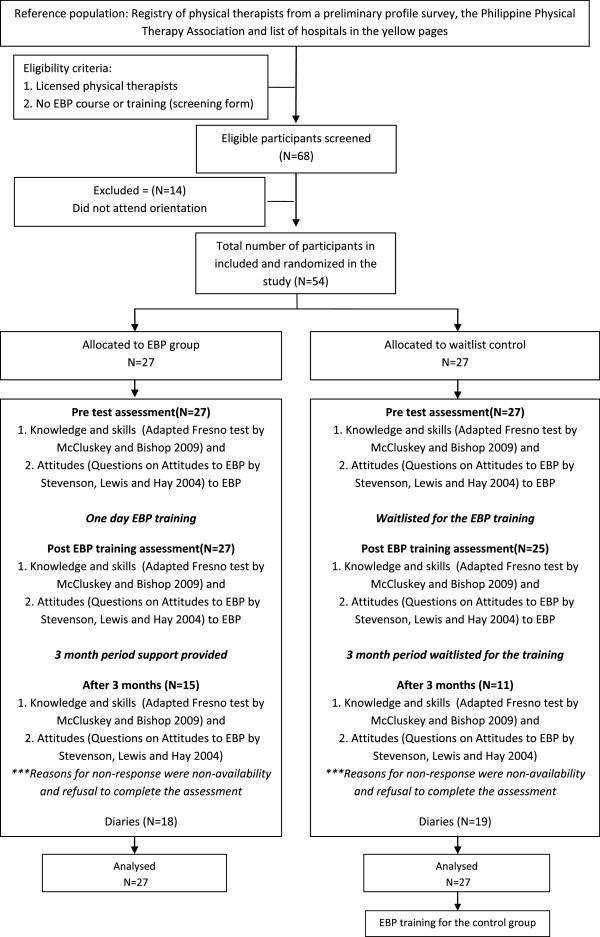
Consort diagram.

Sixty-eight potentially-eligible physical therapists expressed interest in taking part in the study. Fourteen did not attend the orientation and were not included in the random allocation process. A total of 54 physical therapists were randomized to the EBP training group (N = 27) and the waitlist control (N = 27) groups. Fifty four (54) physical therapists completed the pre-training assessments, 52 (EBP group = 27, waitlist control group = 25) completed the post training assessments and 26 completed the 3 months post training assessment (EBP group = 15, waitlist control group = 11). Of the 54 physical therapists in the study, 37 returned the behavior diaries (EBP group = 18, waitlist control group = 19). See Figure 1.

The physical therapists were similar at baseline in terms of age and the main study outcome (EBP knowledge and skills measured using the AFT), but different in terms of years of practice (Table 1). There were 12 (44%) females and 15 (56%) males in the EBP group and 17 (63%) females and 10 (37%) males in the waitlist control group. There was no difference in the proportion of females and males in both groups.

**Table 1:** Characteristics of the physical therapists at pre-training

**Characteristics**	**EBP group (N = 27)**	**Waitlist control group (N = 27)**	**P value**
	**(median, IQR)**	**(median, IQR)**	
Age (years)	29.0 (26–36)	28.0 (25–30)	0.05
Years in practice	4.2 (2–7.75)	3.0 (1.13-4)	0.04*
Adapted Fresno Test (AFT) Scores	11.0 (8.0-32.5)	21.0 (5.0-29.5)	0.97

*significant p value; Mann- Whitney U test was used to calculate for differences between groups.

### Study outcomes (EBP domains)

#### EBP Knowledge and skills

**Figure 2:**
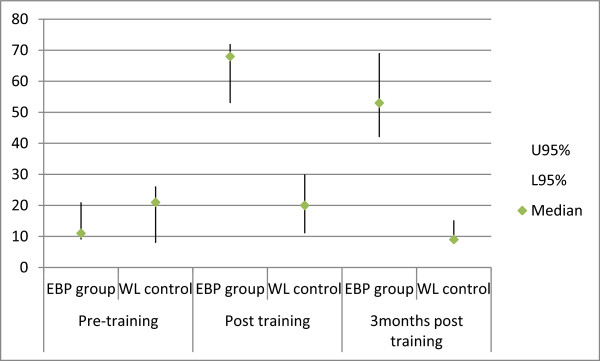
Changes in EBP knowledge and skills of physical therapists.

Improvements in EBP knowledge and skills were found in the EBP group compared with the waitlist control group at immediate post-training and three months after the training, as shown in Table 2 and Figure 2 (using the median scores and 95% confidence interval for the median).

**Table 2:** Changes in EBP knowledge and skills

**AFT scores**	**EBP group (N = 27)**		**Control group (N = 27)**		**P value**
**(total = 0–156)**	**Median, IQR**	**95% CI**	**Median, IQR**	**95% CI**	
Pre training	11.0	9.0-21.0	21.0	7.9-26.1	0.97
	(8.0-32.5)		(5.0-29.5)		
Post training	68.0	53.0-72.0	20.0	11.0-30.0	<0.0001*
	(51.5-76.8)		(9.3-30.8)		
3 months post training	53.0	42.0-69.1	9.0	9.0-15.2	<0.0001*
	(39.2-71.8)		(8.0-27.5)		

*significant p value; Mann- Whitney U test was used to calculate for differences between groups.

The improvement in knowledge and skills of the EBP group was significant in all items of the AFT in over all time periods except for Q2 (Sources of information; advantages and disadvantages) at post training (Table 3).

**Table 3:** Changes in EBP knowledge and skills of the EBP group in each item of the AFT over time periods

**Questions in the AFT**	**Pre training scores**			**Post training**			**3 months post training**		
	**(median, IQR)**			**(median, IQR)**			**(median, IQR)**		
	**EBP group**	**Waitlist control**	**Pre training**	**EBP group**	**Waitlist control**	**Post training**	**EBP group**	**Waitlist control**	**3 months post training**
	**(N = 27)**	**(N = 27)**	**(p value)**	**(N = 27)**	**(N = 27)**	**(p value)**	**(N = 27)**	**(N = 27)**	**(p value)**
Q1 (0–12 points)	5.0 (3.0- 7.8)	5.0 (3.0-6.0)	0.41	10.0 (9.0-12.0)	6.0 (3.0- 8.8)	0.0001*	9.0 (9.0-12.0)	4.0 (3.0-6.8)	0.0001*
Writing a focused question using the PICO									
Q2 (0–24)	6.0 (4.0-8.0)	7.0 (0.0-10.0)	0.87	8.0 (6.0-13.5)	6.0 (2.0-10.0)	0.1036	8.0 (6.0-13)	4.0 (2.0-7.5)	0.0006*
Sources of information; advantages and disadvantages									
Q3 (0–24)	0.0 (0.0-6.0)	0.0 (0.0-5.3)	0.98	12.0 (12.0-12.0)	0.0 (0.0-6.0)	<0.0001*	12.0 (6.0-12.0)	0.0 (0.0-2.3)	0.0005*
Study design that would answer the question									
Q4 (0–24)	0.0 (0.0-2.3)	0.0 (0.0-7.5)	0.48	12.0 (6.0-15.5)	0.0 (0.0-6.0)	0.0001*	8.0 (6.5-14.0)	0.0 (0.0-3.0)	<0.0001*
Search strategy									
Q5 (0–24)	0.0 (0.0-0.0)	0.0 (0.0-0.0)	0.96	5.0 (5.0-14.0)	0.0 (0.0-5.0)	0.0002*	5.0 (0.0-14)	0.0 (0.0-4.5)	0.0038*
How is relevance of the study determined?									
Q6 (0–24)	0.0 (0.0-0.0)	0.0 (0.0-0.0)	0.77	10.0 (5.0-10.0)	0.0 (0.0-0.0)	<0.0001*	5.0 (0.0-10.0)	0.0 (0.0-0.0)	0.0005*
How is validity of the study determined?									
Q7 (0–24)	0.0 (0.0-0.0)	0.0 (0.0-0.0)	0.70	5.0 (5.0-9.0)	0.0 (0.0-0.0)	0.0001*	5.0 (1.3-5.0)	0.0 (0.0-0.0)	<0.0001*
How are magnitude and significance determined?									

*significant p value; Mann- Whitney U test was used to calculate for differences between groups.

#### Attitudes

No statistical difference was found between the EBP group and waitlist control group at pre-training for all but two of the EBP attitudes. This was regarding changing practice if good quality evidence exists, and confidence in undertaking a literature search. Whilst a slightly larger percentage of the EBP group agreed that practice should change if good quality evidence exists, none of the physical therapists (in either group) disagreed. Whilst a larger percentage of the control group agreed they lack confidence in undertaking a literature search, a larger percentage of the EBP group neither agreed nor disagreed, which implies that most of the physical therapists (in either group) were not really confident to do a literature search (Table 4).

**Table 4:** Changes in EBP attitudes

**Attitudes questions**		**Pre-training**			**Post training**			**3 months post training**		
		**EBP**	**Control**	***P value***	**EBP**	**Control**	***P value***	**EBP**	**Control**	***P value***
		**(N = 27)**	**(N = 27)**		**(N = 27)**	**(N = 27)**		**(N = 27)**	**(N = 27)**	
Clinical practice should be based on the best available evidence	Agree	26	27	>0.05	26	26	>0.05	26	27	>0.05
		(96.3%)	(100%)		(96.3%)	(96.3%)		(96.3%)	(100%)	
	Disagree	0	0		0	0		0	0	
	Neither	1	0		1	1		1	0	
		(3.7%)			(3.7%)	(3.7%)		(3.7%)		
We should change our practice if good quality evidence suggests we should	Agree	25	20	<0.05	27	23	>0.05*	26	23	>0.05
		(92.6%)	(74.1%)		(100%)	(85.2%)		(96.3%)	(85.2%)	
	Disagree	0	0		0	0		0	0	
	Neither	2	7		0	4		1	4	
		(7.4%)	(25.9%)			(14.8%)		(3.7%)	(14.8%)	
I would find it difficult to change what I already do in clinical practice	Agree	2	5	>0.05	0	4	<0.05*	2	3	>0.05
		(7.4%)	(18.5%)			(14.8%)		(7.4%)	(11.1%)	
	Disagree							22	16	
								(81.5%)	(52.3%)	
	Neither	8	7		2	4		3	8	
		(29.6%)	(25.9%)		(7.4%)	(14.8%)		(11.1%)	(29.6%)	
I have support form management to undertake EBP	Agree	11	10	>0.05	18	12	>0.05	15	15	>0.05
		(40.7%)	(37.0%)		(66.7%)	(44.4%)		(55.6%)	(55.6%)	
	Disagree	2	2		0	1		4	0	
		(7.4%)	(7.4%)			(3.7%)		(14.8%)		
	Neither	11	15		9	14		8	12	
		(40.7%)	(55.6%)		(33.3%)	(51.6%)		(29.6%)	(44.4%)	
I would lack confidence in undertaking a literature search	Agree	9	17	<0.05*	1	7	<0.05*	0	11	<0.05*
		(33.3%)	(63.0%)		(3.7%)	(25.9%)			(40.7%)	
	Disagree	6	4		25	14		26	9	
		(22.2%)	(14.8%)		(92.6%)	(51.6%)		(96.3%)	(33.3%)	
	Neither	12	6		1	6		1	7	
		(44.4%)	(22.2%)		(3.7%)	(22.2%)		(3.7%)	(25.9%)	
I would feel confident in undertaking a critical appraisal	Agree	2	2	>0.05	24	14	<0.05*	24	9	<0.05*
		(7.4%)	(7.4%)		(88.9%)	(51.6%)		(88.9%)	(33.3%)	
	Disagree	17	13		0	6		0	9	
		(63.0%)	(48.1%)			(22.2%)			(33.3%)	
	Neither	8	12		3	7		3	9	
		(29.6%)	(44.4%)		(11.1%)	(25.9%)		(11.1%)	(33.3%)	

*significant p value; Chi-Square test of proportion was used to calculate for p values.

At immediate post-training and three months post-training, significant differences were found between the groups in terms of attitudes, specifically in terms of the following EBP activities:

1. Lacking confidence in undertaking a literature search (more physical therapists disagreed to this in the EBP group than the control group) and

2. Confidence in undertaking a critical appraisal (more physical therapists agreed to this in the EBP group than the control group).

Interestingly, it could be noted that there was also an increase in number in these attitudes items in the control group at post training period. Possible reasons are explored in the discussion section.

#### EBP behavior

A total of 37 physical therapists submitted their diaries for analysis (EBP group =18, waitlist control group =19). Of the 18 diaries from the EBP group, 16 had reported cases (unique cases = 14, usual cases = 9) and 2 did not contain any documented activity. Of the 19 diaries from the waitlist control group, 13 had reported cases (unique cases = 10, usual cases = 10) and 6 did not contain any documented activity. For the purpose of reporting, the activities were categorized as to EBP behaviors (formulating PICO, logging PICO, searching research evidence, appraising evidence and applying the evidence) and non-EBP behaviors (asking colleagues, asking medical doctors and reading textbooks).

a. Unique cases

Fourteen physical therapists from the EBP group and ten from the waitlist control group reported to have had unique cases. For unique cases seen, more physical therapists in the EBP group significantly performed EBP behaviors (such as formulating their PICO, logging a PICO, searching for research evidence, appraising and applying the evidence) compared with the waitlist control group. More physical therapists in the waitlist control group significantly performed non-EBP behaviors (such as asking medical doctors and reading textbooks). No difference was noted between groups in terms of asking colleagues (Table 5).

b. Usual cases

Nine physical therapists from the EBP group and ten from the waitlist control group reported to have had usual cases. For usual cases seen, more physical therapists in the EBP group significantly performed EBP behaviors (such as formulating their PICO, searching for research evidence, appraising and applying the evidence) compared with the waitlist control group. No difference was noted between groups in terms of the non-EBP behaviors (asking colleagues, asking MDs and reading textbooks (Table 6).

**Table 5:** Comparison of behavior performed by the physical therapists when faced with new or unique cases

	**EBP group (N = 14)**	**Waitlist control group (N = 10)**	**P value**
***EBP behaviors when faced with new/unique cases***			
Physical therapists who formulated PICO	11 (78.6%)	0 (0%)	0.0001*
Physical therapists who logged PICO	4 (28.6%)	0 (0%)	0.037*
Physical therapists who searched research evidence	10 (71.4%)	3 (30%)	0.04*
Physical therapists who appraised evidence	4 (28.6%)	0 (0%)	0.037*
Physical therapists who applied evidence	4 (28.6%)	0 (0%)	0.037*
***Non-EBP behaviors when faced with new/unique cases***			
Physical therapists who asked colleague	11 (78.6%)	8 (80%)	0.93
Physical therapists who asked MD	6 (42.9%)	10 (100%)	0.003*
Physical therapists who read textbooks	8 (57.1%)	10 (100%)	0.017*

*significant p value; Chi-Square test of proportion was used to calculate for p values.

**Table 6:** Comparison of behavior performed by the physical therapists when faced with usual cases

	**EBP group (N = 9)**	**Waitlist control group (N = 10)**	**P value**
***EBP behaviors when faced with usual cases***			
Physical therapists who formulated PICO	8 (88.9%)	0 (0%)	0.00009*
Physical therapists who logged PICO	2 (22.2%)	0 (0%)	0.16
Physical therapists who searched research evidence	9 (100%)	3 (30%)	0.0016*
Physical therapists who appraised evidence	3 (33.3%)	0 (0%)	0.04*
Physical therapists who applied evidence	4 (44.4%)	0 (0%)	0.018*
***EBP behaviors when faced with usual cases***			
Physical therapists who asked colleague	7 (77.8%)	8 (80%)	0.906
Physical therapists who asked MD	4 (44.4%)	7 (70%)	0.26
Physical therapists who read textbooks	7 (77.8%)	7 (70%)	0.70

*significant p value; Chi-Square test of proportion was used to calculate for p values.

## Discussion

This is the first known randomized controlled trial to be undertaken in the field of allied health, in terms of assessing the effects of a carefully designed, context-specific EBP training program which measured change in all EBP domains of knowledge, skills, attitudes and behavior. This is also the first study to assess the impact of an EBP training intervention among physical therapists in a developing county, the Philippines.

The EBP training program delivered to the physical therapists was developed from a series of preliminary studies that informed the design and components of the intervention. The program resulted in improvements in EBP knowledge, skills, attitudes and behavior (evidence seeking) immediately after the training and over a three month period of providing support and observation. However, these findings need to be interpreted with caution due to the number of drop outs in the three months post training assessment.

### Changes in EBP knowledge and skills

The EBP training program resulted in significant improvement in EBP knowledge and skills at post-training. At three months post training, whilst there were also improvements in these domains, it can be noted that there were fewer physical therapists assessed at this point, thus the improvements reported may not exactly reflect the true effect of the intervention at this point of assessment. Nonetheless, the improvement in these EBP domains could be expected due to the low pre-training scores, and the naïve EBP nature of the participants. The physical therapists in the Philippines who participated in the study had no prior and formal training in EBP, thus, it is expected that after undertaking the training, scores in the knowledge and skills test would improve. The findings of this study are similar to those of other similar studies, which provided EBP training programs to other allied health professionals and assessed the impact of the training in terms of knowledge and skills. These were the studies on occupational therapists [36] and social workers [37] which both reported improvements in EBP knowledge and skills immediately post-training and after three months [37] and eight months follow-up respectively [36]. Any EBP training program delivered to any health professional can result in improved knowledge and skills as there is (presumably) new knowledge being provided to the participants [3,4].

In comparing our study with the findings of a study among occupational therapists which also used the AFT of knowledge and skills [36], participants in our study had lower pre-training scores compared to the occupational therapists. The study on occupational therapists had no eligibility restrictions regarding prior training in EBP, thus, the occupational therapists could have prior training in, or exposure to, EBP resulting to higher pre-training AFT scores. In both studies, knowledge and skills improved post training.

### Changes in EBP attitudes

Physical therapists in both groups had common beliefs regarding underpinning practice with the best evidence and changing practice, if good quality evidence suggests that changes in practice should be made. However, in the post-training assessments, the EBP group was significantly more confident in undertaking literature searching and critical appraisal. These findings occurred as expected as an effect of the EBP training program, wherein the physical therapists in the EBP group had lectures and practical sessions on designing a search strategy and critically appraising the literature. Same results were found at three months post-training. These were further validated in the analysis of the diaries where there were EBP behaviors related to searching for evidence and appraising the evidence. However, due to lesser number of physical therapists assessed at this point, these findings should be interpreted with caution.

The improved attitudes in EBP in this study are similar to other studies on physical therapists (Stevenson et al. 2004) [35], occupational therapists [36] and social workers [37] but contradicting findings from a study of a mixed group of health professionals [1]. The common characteristic of this study and the studies amongst physical therapists, occupational therapists and social workers that might have influenced the improvement in EBP attitudes was that the training provided to the allied health professionals included additional strategies or components (such as opinion leaders and support for the EBP activities), other than just teaching the basic steps to EBP or specific skills such as critical appraisal.

An interesting finding in this study relating to attitudes in EBP was the increase in number (though insignificant) of physical therapists in the waitlist control group at post training who agreed to be more confident in undertaking literature search and critical appraisal. It could possibly be that the physical therapists in the control group attempted to read on these items after the pre-training assessment period and perceived that they are able to perform such activities. It could also be related to social desirability bias, where respondents answer favorably to self report assessments because they know that the answers are socially acceptable or the answers are the target responses [38]. However, in this study, the issue on whether the physical therapists have accessed information on searching and appraising evidence and feel confident in doing such activities, or have exhibited social desirability bias in these attitudes, is addressed and validated by the use of an objective and psychometrically tested Adapted Fresno Test. In Questions 4(searching) and 6 (assessing for validity of the evidence) of the AFT, median scores were zero as there were no answers in the test that could be given points for evidence of knowledge and skills on searching and appraising the evidence among those in the waitlist control group.

### Changes in EBP behavior

From the diaries collected from the physical therapists at three months post training, there were more physical therapists in the EBP group who performed EBP behaviors compared with the waitlist control group. Formulating a clinical question and searching for a research article were the most common EBP behaviors reported by the physical therapists. Logging a PICO was not as common as formulating a question and searching research evidence because the physical therapists only logged their PICO if they needed assistance in searching for the best evidence from the researchers (part of the online support intervention). Most of the physical therapists retrieved their own evidence as seen in the number of those searching for evidence. Formulating a clinical question using PICO, logging PICO as needed and searching for research evidence all relate to evidence-seeking behaviors. Appraising and applying the evidence to an actual patient case, which relates to practice behaviors, were also significantly noted in both groups. However, fewer physical therapists reported to have undertaken these practice behaviors as expected, as the physical therapists were not first contact practitioners. It is necessary to obtain a referring doctor’s approval prior to making changes in prescribed treatment. However, this study indicated that there were some physical therapists that were able to apply the evidence and negotiate treatment choices with the referring doctors and other health professionals, using the EBP checklist. This highlights that it is possible to still apply the evidence in practice despite a cultural discipline-based hierarchy. Thus being non-first contact practitioners should not be a barrier to delivering treatment with the best evidence. However, this scenario could have occurred in practice setting where the organization/department and the health professionals in the department had open discussions regarding patient care. Environmental or organizational context plays a major role in the potential for EBP activities to take place [11].

It was also observed that whilst the physical therapists in the EBP group made more EBP behaviors, they were still adopting non-EBP behaviors such as asking a colleague, a medical doctor and reading textbooks. This is usual practice and therefore arguably difficult to change quickly or sustainably. Changing usual practice behavior takes time, and multiple strategies and ongoing input are required in order to make the change sustainable [11,39-41]. What were mostly addressed in the EBP training provided to the physical therapists were a small number of the EBP domains discussed by Michie et al. [42], these being knowledge, skills, environmental context, social/professional role identity and social norms. There are more domains that need to be considered to fully influence behavior change which are beliefs about capabilities, motivation and goals amongst others [42]. However, whilst the EBP training may have only covered some domains of behavior change, it is pleasing to note that the physical therapists in the EBP group had more EBP-behaviors than non-EBP behaviors, compared with the control group.

It is important to note that whilst there were more EBP behaviors made by the EBP group in this study, the findings reflect only those physical therapists whose diaries were available for analysis. Diaries which were not available or not returned for analysis may have a similar, or different set of behavior.

### Limitations of the study

The sampling frame and the number of drop outs especially at the three months post training assessment constrains making a general conclusion about the impact of the EBP training for physical therapists in the Philippines. However, there is an indication of the improved outcomes of targeted EBP training but only to the sample in this study from which the outcomes were obtained. Data regarding reasons for drop out or non-compliance was not available in this study but may be good to collect in future studies to further understand behaviors of the physical therapists and inform the design of training programs in the future.

### Implications for practice

On the findings of this study, physical therapists can benefit from the training in terms of knowledge, skills, attitudes and behavior in EBP. Lack of awareness and lack of knowledge and skills in EBP are identified barriers especially in developing countries. The model of EBP training in this study has the potential to address these to barriers which may be regarded as “baby steps” to getting evidence into practice and may not translate to improved EBP behavior in most cases, but is still worth having as an initial platform to build EBP capacity.

### Implications for research

This model of EBP training program has been tested among physical therapists who were not first contact practitioners and there is some benefit in its use. Future research needs to focus on targeting more individuals to improve the sampling frame and improving intervention strategies and monitoring to complete post test measurements. The EBP training program can also be modified as required, delivered and then evaluated in context in future studies.

## Conclusion

This training program took the form of a complex intervention, by providing context-specific EBP training program for Filipino physical therapists (EBP for FilPTs) which can be modified as needed by local context. There were improved outcomes of EBP domains immediately post training, and to some extent, at three months post training. However, findings still need to be interpreted with caution due to the limitations of the study.

## Competing interests

The authors declare that they have no competing interests.

## Authors’ contributions

All authors were responsible in conceptualization of the study design, analysis of the results and writing of the paper. JD was responsible for designing and carrying out the intervention (EBP training program) with significant input from KG and SK. All authors read and approved the final manuscript.

## Pre-publication history

The pre-publication history for this paper can be accessed here:

http://www.biomedcentral.com/1472-6920/14/147/prepub

## Supplementary Material

Additional file 1 EBP Checklist. Click here for file
