# Supplementary material for: Effectiveness of the tailored Evidence Based Practice training program for Filipino physical therapists: a randomized controlled trial
Source: BMC Med Educ. 2014 Jul 17;14:147. doi: 10.1186/1472-6920-14-147 (PMC4131475; doi:10.1186/1472-6920-14-147)
Supplement: Additional file 1 — EBP Checklist. [file 1472-6920-14-147-S1.docx]

**EBP Checklist**

**RECOMMENDATION FOR APPLYING THE FINDINGS**

| **Clinical relevance or Applicability**   - Is the population similar to your patient case and values? - Was the intervention described in detail for you to replicate? - Do you have the skills and facilities (equipment/venue) to apply the intervention? | **YES** | **NO** |
| --- | --- | --- |
| **Validity of the evidence-base**   - Is the study valid based on the results of the appraisal? - Were outcome measures valid? - Were other relevant factors considered? | **YES** | **NO** |
| **Magnitude of effects or Clinical Impact**   - Are findings clinically significant? - What are the expected effects? | **YES** | **NO** |
| **Applicability**  Based on the clinical relevance, validity and magnitude of effects, are you confident to apply the findings to your own patient? | **YES** | **NO** |
| **Barriers to applying the evidence**  Given that the findings are clinically relevant, valid, expected effects are reproducible and applicable, what would make it difficult for you to apply the findings to your patient? | *List potential barriers*  *1.*  *2.*  *3.*  *4* | |
| **Strategies to address barriers**  What are your suggestions to address the barriers? (please identify realistic and doable strategies) | *List possible strategies*  *1.*  *2.*  *3.*  *4.* | |
